# Perinatal Outcomes Associated With the Modified Shirodkar Cervical Cerclage Technique

**DOI:** 10.7759/cureus.62924

**Published:** 2024-06-22

**Authors:** Ramiro Hidalgo Yánez, Diana M Solorzano Alcivar, Santiago Chavez Iza

**Affiliations:** 1 Obstetrics and Gynecology, Pontificia Universidad Católica del Ecuador, Quito, ECU; 2 Obstetrics and Gynecology, Hospital de Especialidades Carlos Andrade Marín, Quito, ECU

**Keywords:** shirodkar stitch, cervical incompetence, shirodkar cerclage, shirodkar modified technique, cervical cerclage

## Abstract

Objective: The objective of this study was to describe demographic and clinical characteristics and surgical and neonatal results related to the modified Shirodkar cervical cerclage technique.

Materials and methods: This was an observational descriptive and retrospective study. Data was called from anonymized medical records of women who were pregnant and diagnosed with cervical incompetence and who had also undergone cervical cerclage procedures using the modified Shirodkar technique. The variables recorded included demographics such as the maternal age of patients, clinical features like obstetric history, physical examination, and ultrasound findings, and surgical and neonatal outcomes. The qualitative variables were processed using frequencies and percentages, and the quantitative variables were obtained through median, interquartile range, mean, and standard deviation.

Results: Our study included 39 anonymized medical records. The main indication for cervical cerclage placement was prophylactic (56%). The median gestational age at cerclage placement was 16 weeks, with a median gestational age at birth of 38 weeks; only 13% had complications related to prematurity, and 5% were admitted to the neonatal intensive care unit.

Conclusion: The modified Shirodkar technique is associated with favorable surgical, maternal, and neonatal outcomes.

## Introduction

Preterm labor is an important complication in ​​obstetrics and neonatology in both developed and developing nations, with a preterm birth rate of about 4-16% [1], It is associated with critical neonatal complications such as respiratory distress syndrome, intraventricular hemorrhage, necrotizing enterocolitis, and retinopathy of prematurity; depending on the degree of affectation, it can leave permanent consequences that affect the neurological and cognitive development of the newborn. Additionally, it has a substantial economic impact due to the prolonged hospitalization and emotionally affects the family [2].

According to Bloomfield et al., cervical insufficiency is a significant cause of preterm birth, present in 0.5% of all pregnancies [3]. As Vink and Feltovich mention, this clinical condition refers to a primary dysfunction of the cervix resulting in an inability to maintain gestation [4]. This condition manifests as cervical dilatation and shortening without pain before 37 weeks of pregnancy in the absence of labor, placental abruption, or chorioamnionitis [5].

The causes of this condition are cervical trauma, especially iatrogenic, which can be secondary to injuries during labor; previous surgeries such as cervical conization, uterine and cervical malformations, some more rare causes, are collagenopathies such as Ehler Danlos syndrome and Marfan syndrome and, in certain cases, it can be idiopathic [6]. 

According to the American College of Obstetrics and Gynecologists (ACOG), cervical cerclage should be considered in patients with a history of previous miscarriage or preterm birth in the absence of labor or abruptio placentae, especially if the woman has had more than one occurrence [7]. It should also be considered in pregnant women who show cervical dilatations without symptoms. Additionally, cervical cerclage may be recommended for patients with a history of previous losses and who are currently pregnant with a singleton pregnancy and have a cervical length shorter than 25 mm. Accurate management can increase the chances of carrying a full-term pregnancy, which also reduces prematurity and its consequences. However, cerclage is not recommended for women pregnant with twins because it can increase the risk of preterm birth. ACOG suggests routinely measuring cervical length in all pregnant women as a screening method for preterm birth to identify a short cervix, which is defined as a cervical length less than 25 mm before 24 weeks of pregnancy [8].

Cervical cerclage is a surgical procedure that involves the placement of a non-absorbable suture or tape, which is inserted at the level of the cervix with the purpose of providing support and preventing premature delivery. It can be done prophylactically, also known as history-indicated cerclage, therapeutically or ultrasound-indicated cerclage, and emergency or physical examination-indicated cerclage [9]. 

Contraindications for placing a cervical cerclage are active preterm labor, chromosomal or anatomical fetal malformations, uterine bleeding of unknown cause, and clinical evidence of chorioamnionitis: spontaneous premature rupture of membranes [9]. 

Several surgical techniques are available to perform a cerclage; the best known are Shirodkar and McDonald. The initial method involves making two incisions and detaching the bladder and rectum, after which a Mersilene band is inserted through the anterior incision to encircle the cervix, the knot is positioned at the 6 o ‘clock position and incisions are stitched [10]. Previous studies using modifications have achieved gestational ages ranging from 36 to 38 weeks [3,11].

In this study, we are proposing a modified Shirodkar technique. This technique is an adaptation of the original procedure that has been carried out by Hidalgo Ramiro and the medical team of the delivery room area at the Hospital de Especialidades Carlos Andrade Marin located in Ecuador.

## Materials and methods

This was a retrospective, descriptive, and observational study without a control group conducted at Hospital de Especialidades Carlos Andrade Marín, Quito, Ecuador. The study was approved by the Committee on Ethics and Research on Human Subjects of Pontificia Universidad Católica del Ecuador (approval number: EO-169-2022, V2) and established that this study did not require consent. 

Study population

Data were collected from anonymized medical records of pregnant women diagnosed with cervical insufficiency and who underwent cervical cerclage using the modified Shirodkar technique in a tertiary hospital, Hospital de Especialidades Carlos Andrade Marín, located in Quito, Ecuador, from August 2017 to August 2022.

Inclusion and Exclusion Criteria

We included anonymized records of participants who were between 14 and 26 weeks of gestation with clinical or ultrasound diagnosis of cervical insufficiency and who had undergone a cerclage using the Shirodkar modified technique by Hidalgo Ramiro. The following exclusion criteria were: incomplete surgical, neonatal, or maternal data and multiple pregnancies. 

The diagnostic criteria for cervical insufficiency included pregnant women with risk factors and recurrent miscarriages ≥1, as well as those with a cervical length measuring less than 30 mm on transvaginal ultrasound or exhibiting cervical dilation upon physical examination in the absence of labor.

Variables

The quantitative variables were age, number of previous losses in pregnancy, cervical length before cerclage placement, cervical dilatation before the procedure, gestational age at cervical placement, operation duration, perioperative bleeding, length of stay after the procedure, gestational age at cerclage removal and at the end of pregnancy, newborn weight at birth, one and five minutes APGAR score.

The qualitative variables were trimester in which the last pregnancy loss occurred, history of cervical cerclage, history of cervical conization, funneling and sludge in ultrasound, indication for cerclage placement in the current pregnancy, antibiotic prophylaxis before cerclage, complications related to the procedure, type of anesthesia, complications related to anesthesia, clinical conditions in newborn related to prematurity, and newborn admitted to the ICU.

Proposed modification of the Shirodkar technique

Shirodkar's original technique requires two transverse incisions of 2 cm, one located at the 12 o'clock position and the other at 6 o'clock. The bladder and the rectum are released, and then a band of Mersilene (Ethicon, Inc., Raritan, New Jersey, United States) enters via the anterior incision and exits via the posterior incision on the right and left. The cervix is surrounded by the band and the knot is made at 6 o'clock, and the incisions are sutured with Vicryl 2-0 (Ethicon, Inc.) [10].

**Figure 1:**
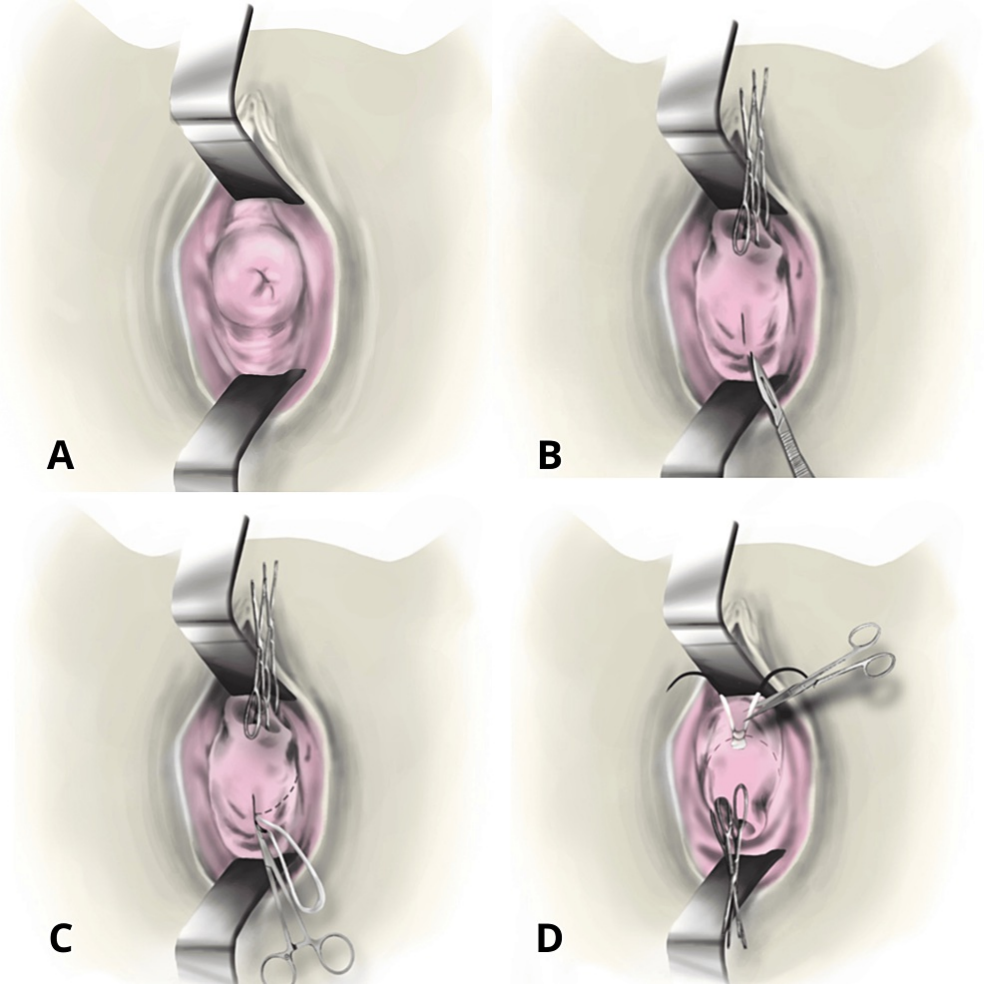
Shirodkar technique modified by Hidalgo (proposed) Original Image, credits: Hidalgo, Solórzano, and Chavez

**Figure 2:**
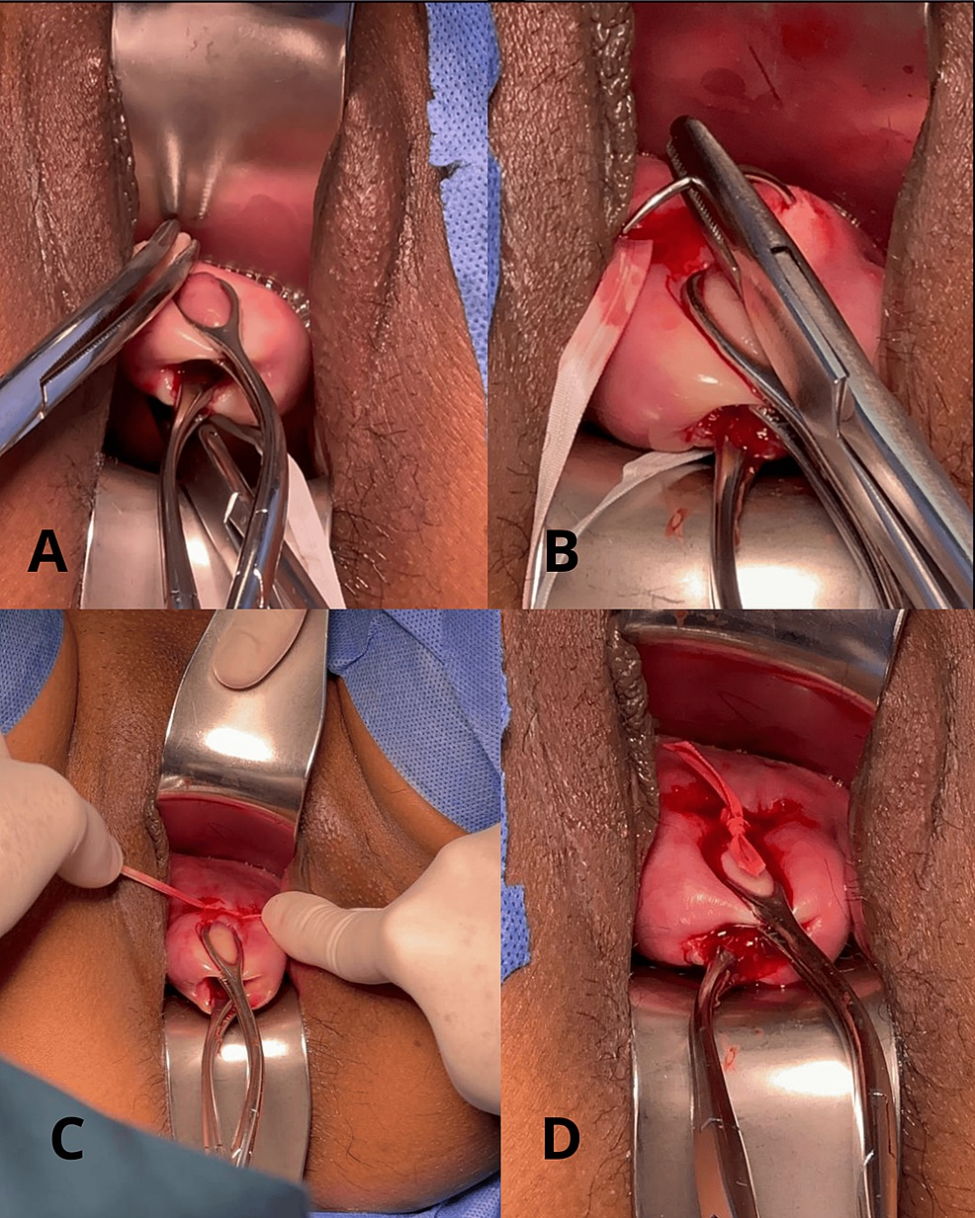
Shirodkar technique modified by Hidalgo Ramiro, MD

The modified procedure was performed in a tertiary hospital by Ramiro Hidalgo, a gynecologist with over 15 years of experience. In this variant of the Shirodkar technique, the patient must be in a lithotomy position and several steps are omitted. It is performed in the following way: after application of asepsis and antisepsis techniques, an anterior and a posterior retractor is placed (Figure 1A), then traction of the cervix is performed with two Foerster forceps (ring forceps), one on the anterior lip and the other on the posterior lip of the cervix. A small longitudinal opening of approximately 3 mm is made in the cervical mucus at 6 o'clock, close to the rectus-vaginal fold (Figure 1B). The “cervix set” band is passed from left to right and from right to left through the opening described above, making the needle travel the entire path in the submucosa of the cervix (Figures 1C, 2A, 2B). The needles come out at 12 o'clock, closest to the vesicovaginal fold; the knot remains at this level, and the thread is cut 1 cm from the knot. Hemostasis is checked, and the retractors are removed (Figures 1D, 2C, 2D).

Statistical analysis

A template was created using Excel (Microsoft Corporation, Redmond, Washington, United States), containing the variables to be studied. The information from the anonymized records was registered and then the Epi Info™ 7.2.5.0 program (Centers for Disease Control and Prevention, Atlanta, Georgia, United States) was used for statistical processing. Frequency and percentages were calculated for the qualitative variables and for quantitative variables, we ran the Shapiro-Wilks test to establish the data distribution, for data that follows a normal distribution, we calculated the mean and standard deviation (SD), while for data with a non-normal distribution, we calculated the median and interquartile range (IQR).

## Results

The diagnostic criteria for cervical insufficiency included pregnant women with risk factors and recurrent miscarriages ≥1, as well as those with a cervical length measuring less than 30 mm on transvaginal ultrasound or exhibiting cervical dilation upon physical examination.

During the period under study, 50 cerclages were placed, but only 39 clinical records met the inclusion criteria, and 11 cases were eliminated due to missing data such as delivery outcomes. The median maternal age at the time of cerclage was 33 years (Table 1). Among the clinical characteristics, it was found that only 15% (n=6) had an obstetric history of cerclage, 21% (n=8) had undergone cervical conization and 34 women had previous pregnancy losses, which mainly occurred in the second trimester (71%, n=24) (Table 2). 

**Table 1:** Demographic and clinical characteristics of patients that went through the modified Shirodkar cervical cerclage technique Data given as mean±SD and median (IQR), where IQR comprises first quartile (Q1), third quartile (Q3) IQR: interquartile range, SD: standard deviation

Variables	Results
Age (years; n=39), median (IQR)	33 (31, 36)
Number of previous losses in pregnancy (n=34), median (IQR)	1 (1, 3)
Cervical length before cerclage placement (mm; n=39), median (IQR)	21 (17, 30)
Cervical dilatation before procedure (cm; n=19), mean±SD	1.73 ± 0.53

**Table 2:** Clinical characteristics of patients that went through the modified Shirodkar cervical cerclage technique.

Characteristics	Frequency	Percentage
The trimester in which the last pregnancy loss occurred (N=34)	-	-
I trimester	10	29
II trimester	24	71
III trimester	0	0
History of cervical cerclage (n=39)	-	-
Yes	6	15
No	33	85
History of cervical conization (n=39)	-	-
Yes	8	21
No	31	79
Funneling in ultrasound (n=39)	-	-
Present	12	31
Absent	27	69
Sludge in ultrasound (n=39)	-	-
Present	3	8
Absent	36	92
Indication for cerclage placement in current pregnancy (n=39)	-	-
Prophylactic	22	56
Therapeutic	14	36
Rescue	3	8

It has been estimated that the median gestation age at cerclage placement was 16 weeks, while the median gestation age at cerclage removal was 38 weeks and the median gestational age at birth was 38 weeks, also the median operation duration was 15 minutes (Table 3).

**Table 3:** Surgical and neonatal results related to modified Shirodkar cervical cerclage technique Data given as median (IQR), where IQR comprises first quartile (Q1), third quartile (Q3) IQR: interquartile range

Variables	Results
Gestational age at cervical cerclage placement (weeks), median (IQR)	16 (14.6, 17.2)
Operation duration (minutes), median (IQR)	15 (12, 16)
Perioperative bleeding (mm), median (IQR)	5 (4, 6)
Length of stay after procedure (days), median (IQR)	1 (1, 2)
Gestational age at cerclage removal (weeks), median (IQR)	38 (36.4, 38.4)
Gestational age at the end pregnancy (weeks), median (IQR)	38 (37, 39)
Newborn weight at birth (gm), median (IQR)	3061 (2718, 3299)
1 minute APGAR score, median (IQR)	8 (8, 9)
5 minutes APGAR score, median (IQR)	9 (9, 9)

Among the surgical and neonatal results, it is notable that complications related to the procedure occurred in 5% (n=2) of women. Additionally, only 13% (n=5) of neonates had complications related to prematurity, and just 5% (n=2) were admitted to the neonatal ICU (Table 4).

**Table 4:** Surgical and neonatal results related to Shirodkar modified technique (N=39)

Surgical and neonatal results	Frequency	Percentage
Antibiotic prophylaxis before cerclage placement		
Yes	39	100
No	0	0
Complications related to the procedure		
None	37	95
Bleeding	1	2,5
Infection	1	2,5
Others	0	0
Type of anesthesia		
Spinal	36	92
General	3	8
Complications related to anesthesia		
Post-dural puncture headache	1	2,5
Urinary retention	2	5
Paresthesia	1	2,5
None	35	90
Others	0	0
Clinical conditions in newborn related to prematurity		
Transient tachypnea of newborn	3	8
Extreme prematurity	2	5
None	34	87
Others	0	0
Newborn admitted to the ICU		
Yes	2	5
No	37	95

## Discussion

The purpose of this study was to describe the perinatal outcomes associated with cervical cerclage using the modified Shirodkar technique in patients diagnosed with cervical insufficiency in a tertiary hospital located in Ecuador.

The median gestational age at cerclage placement was 16 weeks, similar to the one reported by Bloomfield et al., which was 14 weeks [3]. However, it was inserted earlier than Deshpande’s research, which established an average of 20.4 weeks [11]. These differences could be due to the type of indication for its placement; prophylactic cerclage is usually inserted between 11 and 14 weeks, and therapeutic and rescue cerclage can be placed up to 24 weeks of gestation and, in some cases, up to 27.6 weeks [9].

The median gestational age at cerclage removal was 38 weeks, which was a better outcome than other studies. According to Bartolo et al., it was removed at 36.5 weeks [12], and Bloomfield et al.’s study registered this at 36 weeks [3]. The median gestational age at birth was 38 weeks, with a success rate of 95% (n=37); only two neonates were delivered before 32 weeks due to severe preeclampsia and chorioamnionitis. These results, compared to those of other investigations, are favorable. For instance, Bloomfield et al. calculated the same median gestation age at birth and a success rate of 92% (n=51), but four of their neonates were born before 24 weeks [3], while in Bartolo et al.'s study, the median gestation age was similar to ours. However, two neonates were born before 32 weeks [12]. These differences between studies may be caused by the maternal characteristics before cerclage placement, such as gestational age at performing the procedure, presence of prolapsed membranes, type of cerclage indication, complications related to the procedure, obstetric conditions that required premature delivery, the technique, and the surgical skills.

Neonatal median weight at birth was 3061 gm and 5% (n=2) of neonates weighed less than 1500 grams, while Bartolo et al. showed a median of 3190 gm, and only 7,1% (n=1) of them had a weight less than 1500 gm [9]. Also, Bloomfield et al. found a median of 2895 gm [3], and Deshpande et al. calculated an average of 2750 gm [11]. These different medians could be explained by demographic differences amongst pregnant women, weight gain throughout pregnancy, the presence of comorbidities, and the gestational age at the time of the delivery. 

In this study, only 13% (n=5) of the neonates showed clinical conditions related to prematurity, such as transitory tachypnea of the newborn and extreme prematurity, with only two requiring the neonatal intensive care unit. According to Bloomfield et al., 22.6% (n=12) of the newborns had clinical conditions associated with prematurity, and 12 of them were admitted to the neonatal intensive care unit [3]. Even though the outcomes were satisfactory in both studies, the difference between complications may be due to additional factors such as associated maternal and neonatal comorbidities.

During the surgery, the median intraoperative period was 15 minutes, including the anesthesia time. However, the required time compared to other studies was shorter, such as the study by Başbuğ and Doğan, which estimated an average performance time during a therapeutic cerclage of 19 minutes [13], while Frieden et al. calculated an average of 18 minutes [14]. Also, the median bleeding was 5 mm, which was less than that of other studies such as Frieden et al.’s which reported 25 mm [14]. These differences may be due to the type of indication when placing cerclage, maternal comorbidities, complications that may arise during surgery, and the surgeon's experience using the technique.

In our study, the primary type of anesthesia used was spinal in 92% (n=36) of the patients. On the other hand, in Bartolo et al.'s study, most of the patients received general anesthesia (71.4%; n=10) [9], and in Bloomfield et al.'s study, two types of anesthesia were used indistinctly [3]. There is no association between the type of anesthesia used and the maternal and neonatal results, according to Ioscovich et al.; both are safe, and the choice depends on the surgical team and the patient’s preferences [15].

On the other hand, complications associated with anesthesia in the present study manifested in 10% (n=4) of pregnant women as post-puncture headache, difficulty urinating, and paresthesia, while Wang et al. reported cases of hypotension in 30.1% (n=47), nausea in 32.1% (n=50), and vomiting in 17.9% (n=28) in their study [16]. These complications could have been secondary to the patient's position, the type of needle, the technique used to insert it, and the administration of medication.

Regarding surgical complications, these occurred in 5% (n=2) of patients and manifested as bleeding and infection. The first was related to difficulty when placing the suture, and the second occurred six weeks after the intervention and was suspected of chorioamnionitis. In Bloomfield et al.'s study, a case of suture displacement was identified, so it was placed again [3]. However, Bartolo et al. reported one (7.1%) case of premature rupture of membranes that occurred the next day after the procedure and, through the administration of antibiotics, reached 35 weeks of gestation [12]. These complications could have been secondary to different factors such as pre-surgical conditions, unfavorable anatomy, the presence of membrane prolapse and undetected vaginal infections, and the expertise of the surgeon applying the technique.

A median post-intervention hospital stay of one day was estimated. In Bloomfield et al.'s study, the procedure was outpatient, with only one pregnant woman admitted due to the cerclage being placed after 23 weeks [3]. Also, in the studies by Deshpande et al. and Perrotin et al., the average hospitalization time was five days [11,17]. These discrepancies may be because there are no differences found between hospitalization and outpatient management when placing a cerclage, as mentioned by Golan et al. so they may also depend on physician preferences and hospital protocols [18].

The advantages of the proposed technique are that it only requires one small incision that is made on the posterior surface of the cervix and not two types of larger incisions as in the conventional Shirodkar technique, the knot remains at 12 o'clock which reduces the risk of vaginal discharge, the incision does not need to be sutured. There are fewer steps compared to the conventional technique. 

The study’s limitations include that it had a small sample size of pregnant women who underwent cervical cerclage with the modified Shirodkar technique, all performed by a single operator in a tertiary hospital, not having a control group using the original Shirodkar technique to compare the results, and a small number of comparative studies with Shirodkar technique modifications for cerclage placement.

We recommend that prospective studies be done in the future using a control group with the original technique.

## Conclusions

Cervical cerclage is an uncommon procedure in the obstetric area. However, indications for its placement have been established, and surgical skill and the use of a good and correct technique can be directly related to favorable neonatal outcomes. 

There is limited literature regarding modifications of the Shirodkar cerclage technique, and there is a lack of universal consensus on care before, during, and after the procedure. The need for prophylactic antibiotics, a requirement for postoperative hospitalization, has yet to be standardized, which can generate discrepancies between studies and results. Also, there is a need for further comparative studies utilizing this technique.
